# Surface Protein Dispersin of Enteroaggregative *Escherichia coli* Binds Plasminogen That Is Converted Into Active Plasmin

**DOI:** 10.3389/fmicb.2020.01222

**Published:** 2020-06-18

**Authors:** Claudia T. P. Moraes, Jonathan Longo, Ludmila B. Silva, Daniel C. Pimenta, Eneas Carvalho, Mariana S. L. C. Morone, Nancy da Rós, Solange M. T. Serrano, Ana Carolina M. Santos, Roxane M. F. Piazza, Angela S. Barbosa, Waldir P. Elias

**Affiliations:** ^1^Laboratory of Bacteriology, Butantan Institute, São Paulo, Brazil; ^2^Laboratory of Biochemistry and Biophysics, Butantan Institute, São Paulo, Brazil; ^3^Laboratory of Applied Toxinology – Center of Toxins, Immune-Response and Cell Signaling (CeTICS), Butantan Institute, São Paulo, Brazil; ^4^Department of Microbiology, Immunology and Parasitology, Federal University of São Paulo, São Paulo, Brazil

**Keywords:** EAEC, dispersin, plasminogen, plasmin, ECM

## Abstract

Dispersin is a 10.2 kDa-immunogenic protein secreted by enteroaggregative *Escherichia coli* (EAEC). In the prototypical EAEC strain 042, dispersin is non-covalently bound to the outer membrane, assisting dispersion across the intestinal mucosa by overcoming electrostatic attraction between the AAF/II fimbriae and the bacterial surface. Also, dispersin facilitates penetration of the intestinal mucus layer. Initially characterized in EAEC, dispersin has been detected in other *E. coli* pathotypes, including those isolated from extraintestinal sites. In this study we investigated the binding capacity of purified dispersin to extracellular matrix (ECM), since dispersin is exposed on the bacterial surface and is involved in intestinal colonization. Binding to plasminogen was also investigated due to the presence of conserved carboxy-terminal lysine residues in dispersin sequences, which are involved in plasminogen binding in several bacterial proteins. Moreover, some *E. coli* components can interact with this host protease, as well as with tissue plasminogen activator, leading to plasmin production. Recombinant dispersin was produced and used in binding assays with ECM molecules and coagulation cascade compounds. Purified dispersin bound specifically to laminin and plasminogen. Interaction with plasminogen occurred in a dose-dependent and saturable manner. In the presence of plasminogen activator, bound plasminogen was converted into plasmin, its active form, leading to fibrinogen and vitronectin cleavage. A collection of *E. coli* strains isolated from human bacteremia was screened for the presence of *aap*, the dispersin-encoding gene. Eight *aap*-positive strains were detected and dispersin production could be observed in four of them. Our data describe new attributes for dispersin and points out to possible roles in mechanisms of tissue adhesion and dissemination, considering the binding capacity to laminin, and the generation of dispersin-bound plasmin(ogen), which may facilitate *E. coli* spread from the colonization site to other tissues and organs. The cleavage of fibrinogen in the bloodstream, may also contribute to the pathogenesis of sepsis caused by dispersin-producing *E. coli*.

## Introduction

Dispersin is a 10.2-kDa positively charged surface protein, which was initially characterized in enteroaggregative *Escherichia coli* (EAEC) prototypical strain 042 (Sheikh et al., 2002). This protein is encoded by the *aap* (anti-aggregation protein) gene and is secreted across the bacterial cell membrane by the enteroaggregative ABC transporter (Aat) system, remaining non-covalently attached to the bacterial surface (Nishi et al., 2003). In EAEC 042 dispersin neutralizes the bacterial cell surface by repelling and projecting the positively charged aggregative adherence fimbriae II (AAF/II), leading to anti-aggregation and dispersal of bacteria on the intestinal mucosa (Velarde et al., 2007). The immunogenic nature of dispersin is evidenced by the seroconversion detected in United States travelers to Mexico (Huang et al., 2008) and in volunteers orally challenged with EAEC 17-2 harboring *aap* (Nataro et al., 1992). In addition, dispersin increases the rate of ciprofloxacin uptake through the bacterial outer membrane of EAEC strains (Mortensen et al., 2013).

The dispersin encoding gene is highly prevalent in EAEC collections of different studies, yet not present in all strains (Czeczulin et al., 1999; Elias et al., 2002; Jenkins et al., 2007; Boisen et al., 2012; Durand et al., 2016; Havt et al., 2017; Hebbelstrup Jensen et al., 2017; Dias et al., 2020; Guerrieri et al., 2020). The presence of *aap* was also detected in EAEC strains capable to cause urinary tract infection (Olesen et al., 2012; Boll et al., 2013; Herzog et al., 2014) and in Shiga toxin-producing EAEC of serotypes O104:H4 and 0111:H21 (Scheutz et al., 2011; Dallman et al., 2012).

Although dispersin has been firstly described in EAEC, the *aap* gene has also been detected in extraintestinal *E. coli* (ExPEC) (Abe et al., 2008; Nazemi et al., 2011; Riveros et al., 2017), and in other diarrheagenic *E. coli* pathotypes, such as diffusely adherent *E. coli* and rabbit-specific enteropathogenic *E. coli* (Monteiro et al., 2009; Srikhanta et al., 2013). Enterotoxigenic *E. coli* harbors an *aap*-like gene (*cexE*), along with the *aat* operon, encoding its transporter system (Crossman et al., 2010).

Adherence of EAEC to biotic and abiotic surfaces is a complex multifactorial phenotype where fimbriae play an important role (Harrington et al., 2006; Croxen et al., 2013; Kalita et al., 2014). AAF/II fimbriae of EAEC 042 can bind to fibronectin, laminin and type IV collagen (Farfan et al., 2008), with the involvement of the major pilin subunit (AafA) in the binding to fibronectin (Izquierdo et al., 2014). Since EAEC is able to bind to extracellular matrix (ECM) (Farfan et al., 2008; Izquierdo et al., 2014), and dispersin is exposed on the bacterial surface and involved in intestinal colonization (Velarde et al., 2007; Harrington et al., 2009), we questioned if this protein could also bind to ECM. Diverse in nature and composition, the ECM is a complex, three-dimensional network mainly composed of glycoproteins, proteoglycans, and fibrous proteins such as collagens. It provides a structural support to the surrounding cells, and regulates multiple cellular functions including adhesion, migration, proliferation, and differentiation (Teti, 1992).

Binding to plasminogen was also investigated due to the presence of conserved carboxy-terminal lysine residues in dispersin sequences, which are responsible for the binding to plasminogen in several bacterial proteins (Lähteenmäki et al., 2001; Bhattacharya et al., 2012). Moreover, some *E. coli* surface-exposed components can interact with this host protease, as well as with tissue plasminogen activator, leading to plasmin production (Lähteenmäki et al., 2001; Bhattacharya et al., 2012). Since plasmin is a protease involved in ECM degradation, these plasminogen receptors may potentially contribute to tissue spread (Parkkinen and Korhonen, 1989; Korhonen et al., 1992; Lähteenmäki et al., 2001; Bhattacharya et al., 2012). Binding to plasminogen has been evidenced for P and S fimbriae (Parkkinen and Korhonen, 1989; Parkkinen et al., 1991; Korhonen et al., 1992). Murein lipoprotein (Lpp), G (F17) fimbriae and curli also act as plasminogen receptors, thus enhancing plasmin production (Sjöbring et al., 1994; Kukkonen et al., 1998; Gonzalez et al., 2015). Outer membrane proteins such as OmpT (Mangel et al., 1994) and glyceraldehyde-3-phosphate dehydrogenase (GAPDH) (Egea et al., 2007), known to act as a moonlighting protein in several bacteria, have also been described as plasminogen receptors. It has been suggested that GAPDH-bound plasmin may contribute to colonization of the intestinal mucosa by enteropathogenic and enterohemorrhagic *E. coli* (Egea et al., 2007).

Based on the molecular characteristics of dispersin described above, we investigated in this study the binding capacity of purified dispersin to ECM and coagulation cascade components and showed that dispersin binds specifically to laminin and plasminogen. Bound plasminogen is converted into its active form, plasmin, which, in turn, cleaves fibrinogen and vitronectin. These new attributes call attention to dispersin as an important virulence factor that may contribute to tissue adhesion and dissemination.

## Materials and Methods

### Bacterial Strains

The prototypical EAEC strain 17-2 (Nataro et al., 1992) was used as template for *aap* amplification by PCR. EAEC 042 (Nataro et al., 1995) and 17-2 were employed as positive controls for dispersin detection by ELISA. *E. coli* HB101 and DH5α (Sambrook et al., 1989) were used as negative controls in PCR and ELISA. A collection of 278 *E. coli* strains isolated from the bloodstream of patients with bacteremia (Santos, 2013; Freire et al., 2020) was also tested for the presence of *aap*. This collection is composed by one strain isolated from each patient of both genders, various ages and clinical conditions, hospitalized at Hospital São Paulo (Federal University of São Paulo, Brazil) between 2000 and 2008. These strains belong to the bacterial collection Enterobacteriales-Extraintestinal-EPM-DMIP maintained by the Department of Microbiology, Immunology and Parasitology, Federal University of São Paulo (Brazil). All strains were kept in Luria Bertani (LB) broth containing 15% glycerol at −80°C.

### Production of Recombinant Dispersin

The *aap* gene was amplified using the primers Disp(F) and Disp(R), described in Table 1, and EAEC 17-2 as template (GenBank accession number: Z32523.1). These primers amplified a 363-bp fragment corresponding to the entire *aap* sequence, containing *Bam*HI and *Hin*dIII sites at the 5′and 3′ends, respectively. DNA template was obtained from one isolated colony of EAEC 17-2 on LB agar, boiled for 10 min in 300 μL of sterile distilled water. The 25 μL PCR mixture contained 2 μL of bacterial lysate, 25 pmol of each primer, 2 mM of dNTPs, 2.5 μL of 10x PCR Buffer (100 mM Tris–HCl, 500 mM KCl, 0.8% Non-idet P40), 2 mM of MgCl_2_ and 1 U of Taq DNA Polymerase (Thermo Fisher Scientific, Waltham, MA, United States). *E. coli* HB101 was used as negative control. The gene amplification cycle was: 35 × 94°C for 1 min, 59°C for 1 min and 72°C for 1 min followed by 7 min at 72°C. Amplified products were visualized by 1% agarose gel electrophoresis stained with UniSafe Dye (Uniscience, Miami Lakes, FL, United States).

**TABLE 1:** Primers employed in this study, corresponding amplicons and annealing temperatures.

Gene	Primers^a^	Amplicon	Annealing temperature	References
*aap* (cloning PCR)^b^	Disp (F) 5′-GGATCCATGAAAAAAATTAAGTTT-3′	363 bp	59°C	This study (GenBank: Z32523.1)
	Disp (R) 5′-AAGCTTTTATTTAACCCATTCGGTT-3′			
*aap* (detection PCR)	*aap* (F) 5′-CTTTCTGGCATCTTGGGT-3′	232 bp	51°C	Czeczulin et al., 1999
	*aap* (R) 5′-GTAACAACCCCTTTGGAAGT-3′			
*aatA*	*aatA* (F) 5′-CTGGCGAAAGACTGTATCAT-3′	630 bp	56°C	Schmidt et al., 1995
	*aatA* (R) 5′-CAATGTATAGAAATCCGCTGTT-3′			
*aatB*	*aatB* (F) 5′-CTTGAGGACATGATTGAAGG-3′	439 bp	50°C	This study (GenBank: FN554767)
	*aatB* (R) 5′-TTTCCTGTTATACTGACCGG-3′			
*aatC*	*aatC* (F) 5′-AGTTGGAAAGACTTCCACTGC-3′	299 bp	50°C	This study (GenBank: FN554767)
	*aatC* (R) 5′-CGGAGAGAAATGATACATTA-3′			
*aatD*	*aatD* (F) 5′-AGTTCTTATGGGTTACTTGG-3′	499 bp	50°C	This study (GenBank: FN554767)
	*aatD* (R) 5′-ATCCCATATTTGTAGTGGAG-3′			
*aatP*	*aatP* (F) 5′-TATCGACCTAAAACGGTAGG-3′	516 bp	50°C	This study (GenBank: FN554767)
	*aatP* (R) 5′-CCTTGGCGTTTAAAGATGTG-3′			
*aggR*	*aggR* (F) 5′-CGATACATTAAGACGCCTAAAG-3′	346 bp	56°C	Andrade et al., 2014
	*aggR* (R) 5′-TCTGATACATTAAATTCATCTGC-3′			
*aaiA*	*aaiA* (F) 5′-CCCACGACCAGATAACG-3′	476 bp	56°C	Andrade et al., 2014
	*aaiA* (R) 5′-TTTTCAGGATTGCCATTAG-3′			
*aaiG*	*aaiG* (F) 5′- GGGAGTGTTTCAGTCTGGAC–3′	782 bp	56°C	Andrade et al., 2014
	*aaiG* (R) 5′-ATTTGTCACAAGCTCAGCAT-3′			
*aggA*	*aggA* (F) 5′-TCTATCTRGGGGGGCTAACGCT-3′	220 bp	57°C	Boisen et al., 2012
	*aggA* (R) 5′-ACCTGTTCCCCATAACCAGACC-3′			
*aafA*	*aafA* (F) 5′-CTACTTTATTATCAAGTGGAGCCGCTA-3′	289 bp	57°C	Boisen et al., 2012
	*aafA* (R) 5′-GGAGAGGCCAGAGTGAATCCTG-3′			
*agg3A*	*agg3A* (F) 5′-CCAGTTATTACAGGGTAACAAGGGAA-3′	370 bp	57°C	Boisen et al., 2012
	*agg3A* (R) 5′-TTGGTCTGGAATAACAACTTGAACG-3′			
*agg4A*	*agg4A* (F) 5′-TGAGTTGTGGGGCTAYCTGGA-3′	169 bp	57°C	Boisen et al., 2012
	*aag4A* (R) 5′-CACCATAAGCCGCCAAATAAGC-3′			
*agg5A*	*agg5A* (F) 5′-AGATGGAAAGCTTGTCATG-3′	401 bp	57°C	Jønsson et al., 2015
	*agg5A* (R) 5′-GTGGTTACGGATATTATC-3′			

*^a^(F), forward primer; (R), reverse primer. ^b^The underlined nucleotides in Disp(F) and Disp(R) sequences correspond to the BamHI and HindIII restriction sites, respectively.*

The amplified *aap* fragment was excised from the agarose gel, purified with Illustra PCR gel band (GE Healthcare, Pittsburg, PA, United States) and initially cloned into pGEM-T Easy vector (Promega, Madison, WI, United States), following the manufacturer’s instructions. Electrocompetent *E. coli* DH5α were transformed with the ligation (Ausubel et al., 1995) and transformants selected on LB agar plates containing 100 μg/mL of ampicillin. One clone (CT1) was selected for subcloning into the pAE expression vector (Ramos et al., 2004). DNA extracted from CT1 was digested with *Pst*I and *Bam*HI and cloned in pAE previously digested with the same restriction enzymes. Electrocompetent *E. coli* BL21-C43 (Miroux and Walker, 1996) were transformed with the ligation and transformants selected on LB agar plates containing 100 μg/mL of ampicillin. One clone (CT2) confirmed by DNA sequencing was selected as the dispersin-expression clone.

An overnight pre-inoculum of CT2 in LB broth was diluted 1:100 in 2YT medium and incubated at 37°C until the OD_600_ of 0.6, when 1 mM of IPTG was added to the culture and incubated for 3 h at 37°C with 150 rpm shaking. All cultures were performed in the presence of 100 μg/mL of ampicillin. Recombinant dispersin was produced in the soluble fraction of 2YT culture, which was obtained by sonication in PBS 0.01 M (pH 7.4) and then concentrated in a spin concentrator of 20 kDa cutoff (Vivaspin 2; Sartorius, Göttingen, Germany).

### Biochemical Characterization of Recombinant Dispersin

After concentration, recombinant dispersin was purified by RP-HPLC and purity was analyzed by mass spectrometry (MALDI-TOF and ESI-TOF) and 15% SDS-PAGE (Laemmli, 1970) followed by Coomassie Brilliant Blue staining (Sambrook et al., 1989). The purified dispersin was further characterized by amino-terminal sequencing (Edman degradation) and circular dichroism.

Samples were analyzed by reverse-phase high performance liquid chromatography (RP-HPLC) using a binary HPLC system (20A Prominence; Shimadzu Co., Kyoto, Japan). Aliquots of each material were loaded in a C18 column (ACE C18, 5 μm; 100 Å, 250 mm × 4.6 mm) in a two solvent-system: (A1) TFA/water (1/999, v/v) and (B1) ACN/water/TFA (900/99/1, v/v/v). The column was eluted at a constant flow rate of 1 mL/min with a 0–100% gradient of solvent B1 over 20 min. The eluates were monitored by a Shimadzu SPD-M20A detector at 214 nm. Fractions were collected manually.

Liquid chromatography–mass spectrometry (LC–MS) analyses were performed using an Electrospray-Ion Trap-Time of Flight (ESI-ITTOF) (Shimadzu Co.) equipped with binary Ultra-Fast Liquid Chromatography system (UFLC) (20A Prominence; Shimadzu Co.). Samples were dried, resuspended in water/formic acid (0.99/0.01, v/v) and loaded in a C18 column (Shimadzu-pack XR-ODS, 2.2 μm; 100 × 3 mm) in a binary solvent system: (A2) water/formic acid (FA) (999/1, v/v) and (B2) ACN/water/FA (900/99/1, v/v/v). The column was eluted at a constant flow rate of 0.2 mL.min^–1^ with a 0–100% gradient of solvent B2 over 20 min. The eluates were monitored by a Shimadzu SPD-M20A PDA detector before introduction into the mass spectrometer, in which the spray voltage was kept at 4.5 KV and the capillary voltage at 1.76 KV, at 200°C. MS spectra were acquired under positive mode and collected in the 80–2000 m/z range. Instrument control, data acquisition, and data processing were performed with LabSolutions (LCMSsolution 3.60.361 vs.; Shimadzu Co.).

Direct infusion ESI mass spectrometric analyses, for accurate molecular mass determination, were performed in an ESI-IT-TOF as described above. Samples were dried and resuspended in 0.1% FA and manually injected in a Rheodyne injector, at a flow rate of 50 μL/min, in 50% B2. Instrument control, data acquisition, and data processing were performed with LabSolutions (LCMSsolution 3.60.361 vs., Shimadzu Co.).

Reverse-phase high performance liquid chromatography fractions were analyzed using a Matrix-Assisted Laser Desorption Ionization – Time of Flight (MALDI-TOF) mass spectrometer (Axima Performance, Shimadzu Co.). One microliter of the sample was co-crystallized with 1 μL of sinapinic acid matrix (a saturated solution prepared in 50% ACN/0.1% TFA) in the sample plate and dried at room temperature. Mass spectrum was obtained in the 200–25,000 mass/charge (m/z) range, in linear positive mode.

N-terminal amino acid sequencing was performed on a Shimadzu automatic protein sequencer (PPSQ-10 System) by the Edman degradation method (Edman and Begg, 1967), following the manufacturer instructions. Briefly, 300 μg of dispersin were employed in the analysis. The identified amino acids were separated by HPLC, and quantification and identification were performed by comparison with a 25 pmol standard (analyzed at the beginning of each sequence). The sequences were compared and aligned using the BLAST-NCBI algorithm (Altschul et al., 1997).

Circular dichroism spectra were obtained on a JASCO J-810 spectropolarimeter (Jasco, Tokyo, Japan) equipped with a Peltier temperature-controlling unit. Prior to analysis, samples were extensively dialyzed in 10 mM phosphate buffer, and spectra were collected at 0.22 mg/mL protein concentration on a 0.1 cm path length cuvette, at 25°C. The spectrum, collected at the wavelength range of 188–260 nm at 50 nm min^–1^, is an average of 10 accumulations. Data were presented in molar ellipticity (Corrêa and Ramos, 2009), as a function of the wavelengths. The secondary structure percentage composition was obtained by deconvolution of the spectrum, using the server Dichroweb (Whitmore and Wallace, 2008), applying the algorithm Selcon3 (Sreerama et al., 1999).

### Production of Polyclonal Antiserum Against Dispersin

Anti-dispersin serum was produced in a female New Zealand white rabbit by inoculating intramuscularly 200 μg of recombinant protein and 5 mg of Al(OH)_3_ as adjuvant. Two boosters were given after 14 and 21 days and the total blood was collected on day 31 to obtain the serum. This protocol was approved by the Ethics Committee on Animal Use of the Butantan Institute (CEUAIB Protocol #731408317). Serum antibody titers were determined by ELISA against 1 μg of purified dispersin and an immunoblotting was performed to confirm recognition of recombinant dispersin by the anti-serum obtained, as follows. Recombinant dispersin (0.25 μg) was analyzed by SDS-PAGE (15%), and proteins were transferred to a nitrocellulose membrane. After blocking with 5% skimmed milk in PBS (pH 7.2) for 18 h at 4°C, the membrane was washed three times with PBS-Tween 0.05% (PBS-T) and incubated with anti-dispersin serum diluted 1:100 in 2.5% skimmed milk in PBS, for 1 h at room temperature with shaking. The membrane was washed three times with PBS-T and incubated with horseradish peroxidase-conjugated goat anti-rabbit IgG (Sigma-Aldrich, St. Louis, MO, United States) diluted 1:5,000 in 2.5% skimmed milk in PBS, for 1 h at room temperature with shaking. The membrane was washed three times with PBS-T and 3,3′-Diaminobenzidine (DAB) substrate solution (0.05% DAB, 0.015% H_2_O_2_, Tris 0.05 M, NaCl 0.15 M, pH 7.6) was added. Distilled water was used to stop the reaction.

### Binding of Dispersin to ECM and Coagulation Cascade Molecules

Binding assays were performed according to the protocol described by Salazar et al. (2014), with some modifications. ELISA plate wells (Nunc-Immuno plate, MaxiSorp surface; Nunc, Roskilde, Denmark) were coated with 1 μg of collagen I, collagen IV, laminin, cellular fibronectin, plasma fibronectin, fibrinogen, plasminogen or BSA (attachment-negative control protein), in 100 μL of PBS. BSA, ECM, and coagulation cascade molecules were purchased from Sigma-Aldrich. The plates were incubated at 4°C for 16–20 h. After incubation, the wells were washed three times with PBS-T, and blocked with 200 μL of BSA 1% for 2 h at 37°C. Recombinant dispersin (1 μg) was added to each ECM coated well and the plate was incubated at 37°C for 1 h and 30 min. After three washes with PBS-T, anti-dispersin (1:100) was added and the plate was incubated at 37°C for 1 h. The wells were washed three times with PBS-T and 100 μL of a 1:10,000 dilution of horseradish peroxidase-conjugated goat anti-rabbit immunoglobulin G (IgG) (Sigma-Aldrich) were added per well and the plate was incubated at 37°C for 1 h. The wells were washed three times with PBS-T and *o*-phenylenediamine (0.04%) in citrate phosphate buffer (pH 5.0) plus 0.01% (wt/vol) H_2_O_2_ was added. The reaction was allowed to proceed for 15 min and was then interrupted by the addition of 50 μL of 4 M H_2_SO_4_. The absorbance at 492 nm was determined in a microplate ELISA reader (Labsystems Uniscience Multiskan EX). Three independent experiments were performed in triplicates for all assays described above. Student’s two-tailed *t* test was used for statistical analysis (GraphPad Prism vs. 7.04). *P* values lower than 0.05 were considered statistically significant.

The role of lysine residues in dispersin-plasminogen interaction was also investigated. ELISA plate wells were coated with plasminogen (10 μg/mL) and incubations were performed as described above, except for the addition of ε-aminocaproic acid (0–10 mM) along with recombinant dispersin (10 μg/mL). Incubation with antibodies was performed as already described.

### Surface Plasmon Resonance Assays

Interactions between plasminogen and dispersin were studied by surface plasmon resonance (SPR) using the BIAcore T100 system, at 25°C. Plasminogen (Sigma-Aldrich) was covalently immobilized on the BIAcore CM-5 sensorchip (carboxylated dextran matrix) according to the manufacturer’s instructions. Briefly, the CM-5 chip was activated with a 1:1 mixture of 75 mg/mL EDC [1-ethyl-3-(3-dimethylaminopropyl)carbodiimide] and 11.5 mg/ml NHS (*N*-hydroxysuccinimide) for 7 min. Plasminogen (3 μM in 10 mM sodium citrate, pH 5.0) was injected over the CM-5 chip for 7 min at a flow rate of 10 μL/min, at 25°C. Remaining active groups on the matrix were blocked with 1 M ethanolamine/HCl, pH 8.5. Immobilization of plasminogen on a CM-5 sensorchip resulted in a surface concentration of 5.0 ng/mm^2^. Protein solutions of dispersin (2.5–120 μM) were prepared in HBS-P buffer (10 mM HEPES, pH 7.4, 150 mM NaCl, 0.005% Surfactant P20, BIAcore) and were injected at a flow rate of 30 μL/min. The non-linear fitting of association and dissociation curves according to a 1:1 model was used for the calculation of kinetic constants (BIAevaluation software, version 2.0.4). Individual experiments were performed four times.

### Plasminogen Activation

Microplate wells were coated with 1 μg of recombinant dispersin or BSA (attachment-negative control protein). After blocking with 3% BSA, plasminogen (20 μg/mL) was added and incubation proceeded for 1 h at 37°C. The wells were washed 3 times with PBS-T to remove unbound plasminogen. Human urokinase plasminogen activator (uPA, Sigma-Aldrich) (3 U/well) and the chromogenic plasmin-specific substrate D-valyl-leucyl-lysine-ρ-nitroanilide dihydrochloride (25 μg/well, Sigma Aldrich) dissolved in PBS were added. In one of the control reactions, 0.25 μg/μL of plasminogen activator inhibitor I (PAI-1) was added. Plates were incubated at 37°C and the absorbance at 405 nm was read after 24 h. Three independent experiments were performed in triplicates. The MntC protein of *Staphylococcus aureus* was used as positive control (Salazar et al., 2014).

### Fibrinogen and Vitronectin Degradation

Recombinant dispersin (1 μg in 100 μL of PBS – 10 μg/mL) or BSA were immobilized onto microplate wells. Purified MntC protein was used as positive control for fibrinogen cleavage (Salazar et al., 2014). Coated wells were blocked with 3% BSA diluted in PBS and plasminogen (10 μg/mL) was added. Incubation proceeded for 1 h at 37°C. After washing three times with PBS-T, human fibrinogen (10 μg) or human native vitronectin (1 μg) (Sigma-Aldrich) and plasminogen activator uPA (3U) were added. Reaction mixtures were incubated for 0, 1, or 4 h at 37°C, proteins were separated by 12% SDS-PAGE, and transferred to a nitrocellulose blotting membrane. Membranes were blocked with 5% skimmed milk for 1 h at room temperature with shaking, and the immunodetection was performed as described above for immunoblotting for dispersin detection, using primary antibodies anti-fibrinogen (Cloud-Clone, Katy, TX, United States) and anti-vitronectin (Complement Technology, Tyler, TX, United States) diluted 1:1000 and 1:5000, respectively, in 2.5% skimmed milk in PBS. Fibrinogen and vitronectin degradation products were visualized by ECL Analysis System (GE Healthcare) in the UVITEC Cambridge Image System (Alliance vs. 6.7).

### Detection and Sequencing of *aap* in *E. coli* Strains Isolated From Bacteremia

The presence of *aap* was assessed by PCR in 278 *E. coli* strains isolated from the bloodstream of patients with bacteremia. These strains were initially grown on MacConkey agar and subcultured on LB agar, incubated for 18 h at 37°C. DNA templates were obtained from one isolated colony on LB agar, boiled for 10 min in 300 μL of sterile distilled water.

PCR was performed using the *aap*(F) and *aap*(R) primers (described in Table 1) to amplify a 232-bp intern fragment. The 25 μL PCR mixture contained 2 μL of lysate, 25 pmol of each primer, 2 mM of dNTPs, 2.5 μL of 10x PCR Buffer (100 mM Tris–HCl, 500 mM KCl, 0.8% Non-idet P-40), 2 mM of MgCl_2_ and Taq DNA Polymerase (1 U) (Thermo Fisher Scientific). EAEC 17-2 and *E. coli* HB101 were used as positive and negative controls, respectively. Gene amplification cycle was: 35 × 94°C for 1 min, 51°C for 1 min and 72°C for 1 min, followed by 8 min at 72°C. Amplified products were visualized by 1% agarose gel electrophoresis stained with UniSafe Dye (Uniscience) and purified by using the Wizard SV Gel and PCR clean-up system (Promega). Sanger sequencing was performed at the Human Genome and Stem Cell Research Center (HUG-CELL), Institute of Biosciences, University of São Paulo (Brazil). Sequence analyses were performed using BioEdit vs. 7.0.5.

### Dispersin Production by *aap*-Positive *E. coli* Strains

After the screening for the presence of *aap* in the 278 *E. coli* strains isolated from the bloodstream of patients with bacteremia, 8 *aap*-positive and 7 *aap*-negative strains were selected for ELISA assays to detect dispersin. These selected strains were grown in 3 mL of LB broth for 18 h at 37°C with shaking (150 rpm). Bacteria were centrifuged at 2,500 × *g* for 5 min at 4°C. Pellets were suspended in 500 μL of carbonate/bicarbonate buffer (pH 9.6) and 100 μL of each sample were added to ELISA plate wells (Nunc-Immuno plate, MaxiSorp surface; Nunc), in triplicates. After 18 h, coated wells were blocked with 5% of skimmed milk in PBS (pH 7.2) for 2 h at room temperature. Wells were washed three times with PBS-T and 100 μL of anti-dispersin serum diluted 1:200 in PBS were added. Incubation proceeded for 1 h at room temperature. The wells were washed three times with PBS-T and 100 μL of a 1:10,000 dilution of horseradish peroxidase-conjugated goat anti-rabbit IgG (Sigma-Aldrich) were added to each well, followed by incubation for 1 h at room temperature. The wells were washed three times with PBS-T and 100 μL of *o*-phenylenediamine (0.04%) in citrate phosphate buffer (pH 5.0), containing 0.01% of hydrogen peroxide, were added. The reaction was allowed to proceed for 15 min and interrupted by adding 50 μL of 4 M H_2_SO_4_. The absorbance at 492 nm was determined in a microplate reader (Labsystems Uniscience Multiskan EX). The positive controls were EAEC strains 17-2 and 042, and recombinant dispersin protein (1 μg/well), while *E. coli* DH5α and 7 *aap*-negative strains were used as negative controls to determine the cut-off value of absorbance for samples to be considered as positive for dispersin expression. The blank reaction was the recombinant dispersin incubated with the same reagents in the absence of the primary antibody. Three independent experiments were performed in triplicates. Non-parametric Student’s two-tailed *t* test and ANOVA were used for statistical analysis with 95% confidence interval (CI) (GraphPad Prism vs. 7.04).

### Detection of EAEC-Related Genes in *E. coli* Strains Harboring *aap*

The presence of the following EAEC genetic markers were searched in those *E. coli* strains isolated from cases of bacteremia that were positive for the presence of *aap*: *aatPABCD* (type I secretion system of dispersin), *aggR* (aggregative virulence regulator), *aaiA*/*aaiG* (AggR-activated island, type VI secretion system), and *aggA*, *aafA*, *agg3A*, *agg4A*, and *agg5A* (pilins of the aggregative adherence fimbriae I, II, III, IV, and V, respectively) (Nataro et al., 1994; Nishi et al., 2003; Dudley et al., 2006; Boisen et al., 2012; Hebbelstrup Jensen et al., 2014; Jønsson et al., 2015). PCRs were separately performed, as described for *aap* detection, employing the primers presented in Table 1. DNA templates were obtained as specified for *aap* detection. Gene amplification cycles were: 35 × (95°C for 1 min, annealing temperature specified in Table 1 for 1 min and 72°C for 1 min), followed by 7 min at 72°C. Amplified products were visualized by 1% agarose gel electrophoresis stained with UniSafe Dye (Uniscience).

### HEp-2 Cells Adherence Assay

The adherence patterns of *aap*-positive *E. coli* strains were determined following the protocol of Scaletsky et al. (1984). Briefly, strains were grown statically in 3 mL of LB broth for 18 h at 37°C. HEp-2 cell plates containing coverslips were prepared 24 h before the test with 10^5^ cells/well in D-MEM high glucose supplemented with 10% of fetal bovine serum (FBS). Plates were washed six times with PBS (pH 7.2) and 960 μL of D-MEM without antibiotics supplemented with 1% of mannose and 2% of FBS were added to each well. An inoculum of 40 μL of each *E. coli* strain culture was added to each well and the microplates were incubated at 37°C for 3 h (3 h-assay) or with an additional incubation for 3 h after tissue culture media change (6 h-assay). After incubation, wells were washed three times with PBS (pH 7.2), the cells were fixed with 1 mL of methanol and stained with May-Grünwald/Giemsa. The coverslips were slide-mounted and observed by light microscopy (1,000 X).

## Results

### Biochemical Characterization of the Recombinant Dispersin

**FIGURE 1:**
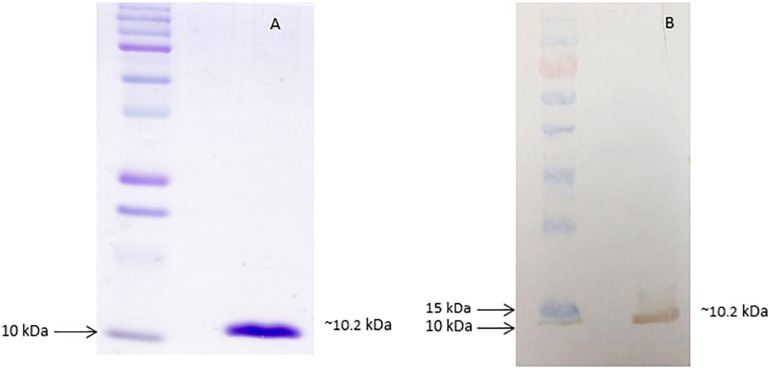
Purified recombinant dispersin. **(A)** SDS-PAGE (15%) of purified recombinant dispersin (∼10.2 kDa) after Coomassie Brilliant Blue staining. Kaleidoscope Protein Standards (Bio-Rad, Hercules, CA, United States) was used as protein size marker. **(B)** Immunoblotting of recombinant dispersin using anti-dispersin serum produced in rabbit (1:100). PageRuler Prestained Protein Ladder (Thermo Fisher Scientific, Waltham, MA, United States) was used as protein size marker.

Since the recombinant dispersin was expressed in the soluble fraction, it was purified from the bacterial lysate supernatant, through molecular weight cut-off separation followed by HPLC (Supplementary Figure S1A), and thoroughly characterized by MS and amino acid sequencing. A unique band was obtained after purification, as depicted in Figure 1A. According to the mass spectrometric analyses, the molecular mass of the obtained protein was 10,120.7 Da (MALDI-TOF/MS) and, more precisely, 10,165.36 (±0.51) Da (ESI-TOF/MS; Supplementary Figure S1B), which is in accordance to the theoretical calculated molecular mass, based on the predicted amino acid sequence of dispersin from prototype EAEC 17-2 (GenBank accession number: Z32523.1) and EAEC 042 (Velarde et al., 2007). Moreover, the measured molecular mass and the amino terminal sequence data demonstrated that the purified recombinant dispersin did not harbor the N-terminal His_6_-tag expected to be generated by cloning into the pAE vector (Ramos et al., 2004). As the dispersin-encoding gene was cloned containing its corresponding signal peptide, the recombinant protein was processed during secretion and the region corresponding to the His_6_-tag and the signal peptide was cleaved. The purified protein matches the mature dispersin from EAEC 17-2, as the twenty N-terminal amino acids were sequenced and determined to be: ^1^GGSGWNADNVDPSQCIKLSG^20^. Structural integrity of the recombinant dispersin was assessed by CD spectroscopy, which showed that the purified protein has, mainly, beta-sheet structures (Supplementary Figure S2), similarly to that obtained by Velarde et al. (2007), describing the structure of dispersin of EAEC 042.

After purification, the recombinant protein was used to obtain polyclonal antibodies in rabbit. The antiserum recognized the purified recombinant protein by immunoblotting analyses (Figure 1B).

### Dispersin Binds to ECM Proteins and Coagulation Cascade Molecules

**FIGURE 2:**
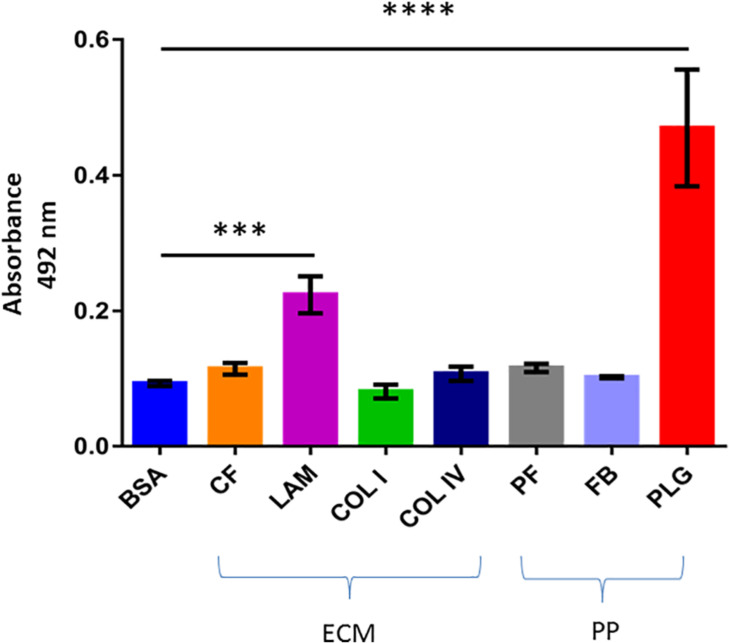
Binding of recombinant dispersin to extracellular matrix (ECM) components and coagulation cascade molecules. ELISA plate wells were coated with 1 μg of ECM components (CF, cellular fibronectin; LAM, laminin; COL I, collagen I; and COL IV, collagen IV) and plasma proteins (PP) (PF, plasma fibronectin; FB, fibrinogen; and PLG, plasminogen). Bovine serum albumin (BSA) was included as negative control. One microgram of recombinant dispersin was added per well. Bound proteins were detected using specific rabbit antiserum to the recombinant protein, followed by peroxidase-conjugated antibodies. Bars represent the mean absorbance value at 492 nm ± standard deviation of three independent experiments, performed in triplicates. For these analyses, Student’s *t*-test was used (***P = 0.0002; ****P < 0.0001).

**FIGURE 3:**
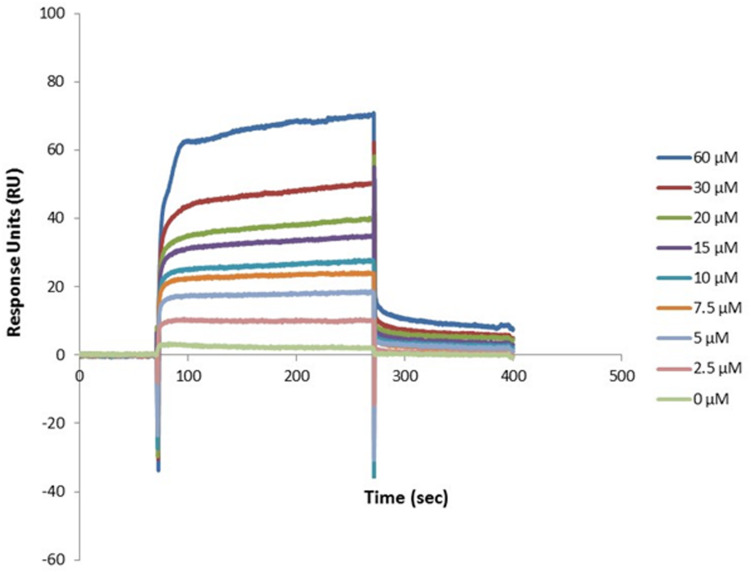
Dose-response binding of recombinant dispersin to purified human plasminogen by surface plasmon resonance (SPR). Interaction of dispersin with plasminogen in the BIAcore T100 system. Dispersin at different concentrations was injected over immobilized plasminogen at a flow rate of 30 μL/min. RU, response units.

We first tested the ability of recombinant dispersin to bind ECM (cellular fibronectin, laminin, and collagens I and IV), and coagulation (plasma fibronectin, fibrinogen, and plasminogen) components in a solid-phase binding assay (Figure 2). Since the binding of dispersin to plasminogen was more pronounced than to the other analyzed proteins (*P* < 0.0001), we investigated further its interaction with dispersin using SPR. As observed in Figure 3, dispersin interacted with immobilized plasminogen in a concentration-dependent manner. Kinetic evaluation of this interaction according to a 1:1 model resulted in the association rate of 5678 ± (4.5 × 10^2^) M^–1^s^–1^ and dissociation rate of 0.0016 ± (4.2 × 10^–5^) s^–1^, which gave an equilibrium dissociation constant (*K*_*D*_) of 0.282 × 10^–6^ M. Although dispersin contains 8% of lysine residues, the interaction with plasminogen was not inhibited in the presence of ε-aminocaproic acid (data not shown).

### Dispersin-Bound Plasminogen Is Converted to Plasmin in the Presence of Urokinase-Type Plasminogen Activator (uPA)

**FIGURE 4:**
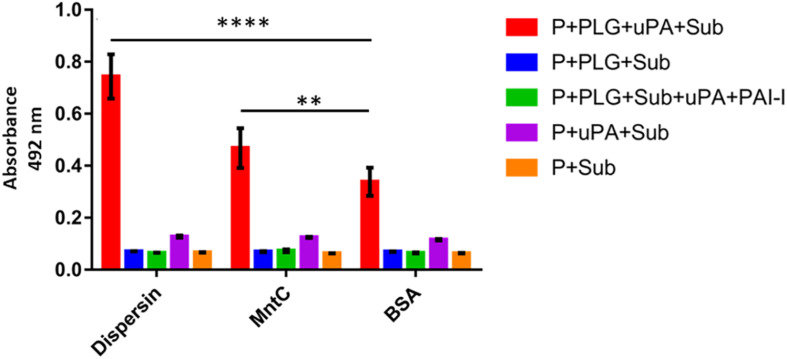
Plasminogen bound to dispersin can be converted in active plasmin. Recombinant dispersin, MntC or BSA (1 μg) were immobilized onto microplate wells and then incubated with plasminogen. After that, uPA (3 U) and the chromogenic substrate for plasmin D-valyl-leucyl-lysine-ρ-nitroanilide dihydrochloride (25 μg/well) were added. Bars represent the mean absorbance value at 492 nm ± standard deviation of three independent experiments, performed in triplicates. For this analysis, Student’s *t*-test was used (**p = 0.0012; ****p = 0.0001). P, dispersin, MntC or BSA; PLG, plasminogen; uPa, human urokinase plasminogen activator; Sub, chromogenic substrate; PAI-1, plasminogen activator inhibitor 1.

The serine protease uPA is a physiological activator that displays fibrinolytic function by converting the proenzyme plasminogen to the active protease plasmin (Danø et al., 1985). Immobilized dispersin was incubated with plasminogen in the presence of exogenously supplied uPA to verify if the dispersin-bound plasminogen could be converted to active plasmin. Plasmin was generated and able to cleave the chromogenic substrate D-valyl-leucyl-lysine-ρ-nitroanilide dihydrochloride (Figure 4). The substrate was not cleaved in the presence of plasminogen activator inhibitor 1 (PAI-1) or in the absence of uPA and/or plasminogen (Figure 4). MntC, a surface protein of *S. aureus* previously shown to cleave the substrate D-valyl-leucyl-lysine-ρ-nitroanilide dihydrochloride through the acquisition of plasmin(ogen) (Salazar et al., 2014), was included as a positive control. Cleavage of above-mentioned substrate was significantly increased in the presence of dispersin or MntC compared to the negative control BSA (*P* = 0.0001 and *P* = 0.0012, respectively).

### Plasmin Bound to Dispersin Cleaves Fibrinogen and Vitronectin

**FIGURE 5:**
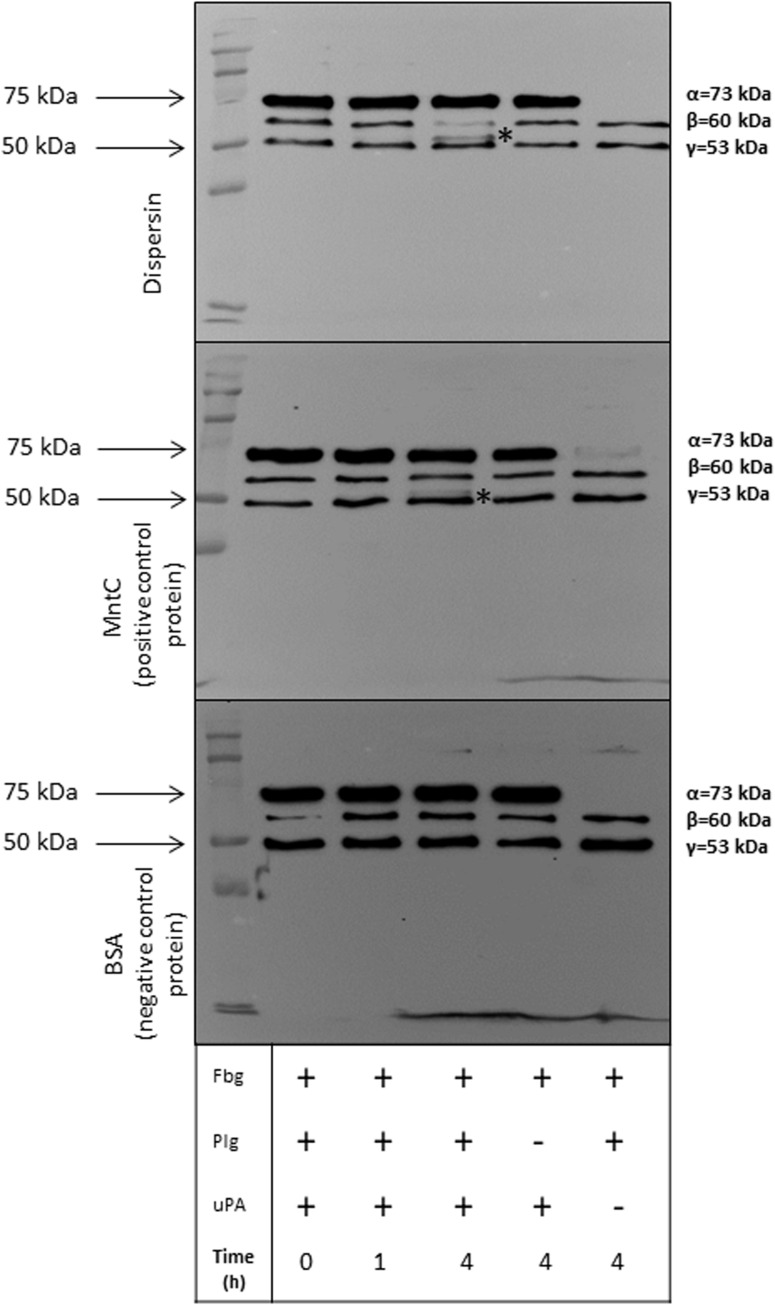
Degradation of human fibrinogen (Fbg) by plasmin(ogen) bound to immobilized dispersin. Recombinant dispersin (10 μg/mL), purified MntC (positive control) and BSA (negative control) were immobilized onto microplate wells and incubated with 10 μg/mL of plasminogen (Plg). After washing, human fibrinogen (10 μg) and plasminogen activator uPA (3 U) were added. After incubation during 1 or 4 h, proteins were separated by SDS-PAGE and transferred to a polyvinylidene difluoride membrane for immunodetection with anti-fibrinogen antibodies (1:1,000) (Cloud-Clone, Katy, TX, United States). Asterisks show fibrinogen cleavage fragments: a band between β and γ chains of fibrinogen was observed in presence of dispersin and MntC, but was not observed in the presence of BSA (negative control), after 4 h of incubation. Kaleidoscope Protein Standards (Bio-Rad, Hercules, CA, United States) was used as protein size marker.

**FIGURE 6:**
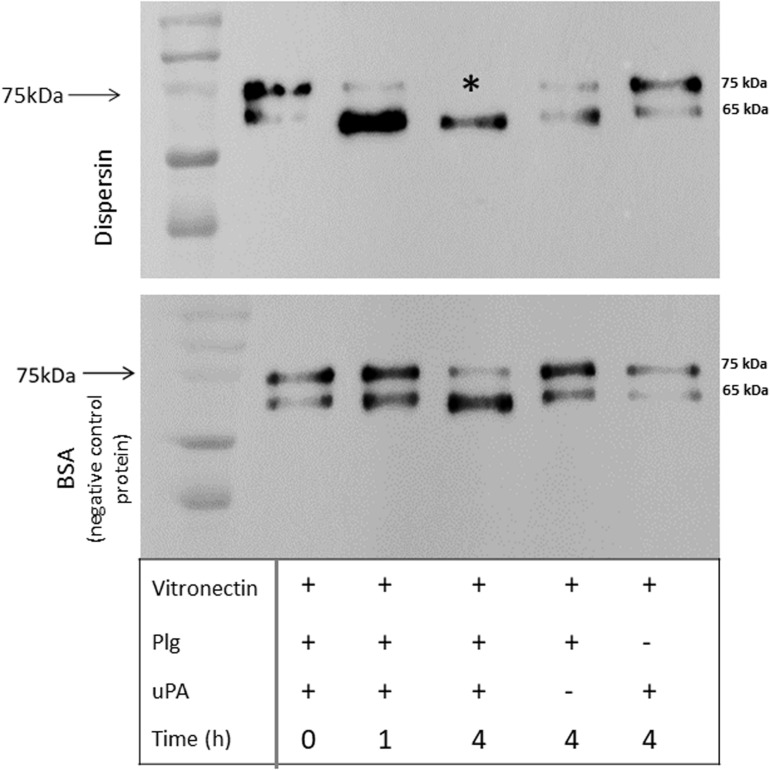
Degradation of human vitronectin by plasmin(ogen) bound to immobilized dispersin. Recombinant dispersin (10 μg/mL) and BSA (negative control) were immobilized onto microplate wells and incubated with 10 μg/mL of plasminogen (Plg). After washing, human vitronectin (1 μg) and plasminogen activator uPA (3 U) were added. After incubation during 1 or 4 h, proteins were separated by SDS-PAGE and transferred to a polyvinylidene difluoride membrane for immunodection with anti-vitronectin antibodies (1:5,000) (Complement Technology, Tyler, TX, United States). Asterisk shows vitronectin degradation: the isoform of 75 kDa was completely degraded in the presence of dispersin, but not in the presence of BSA (negative control), after 4 h of incubation. Kaleidoscope Protein Standards (Bio-Rad, Hercules, CA, United States) was used as protein size marker.

Plasmin plays an important role in fibrinolysis and is also known to degrade a panoply of host components including ECM proteins (Lähteenmäki et al., 2005; Bhattacharya et al., 2012; Sanderson-Smith et al., 2012). We then investigated if dispersin-bound plasmin, besides being able to cleave the chromogenic substrate, could also cleave the physiological substrates fibrinogen and vitronectin. Cleavage of both substrates was detected in a time dependent manner, since cleavage of fibrinogen (Figure 5) and vitronectin (Figure 6) is readily visible after 4 h of incubation but not after 1 h. Degradation of fibrinogen β-chain generated a ∼ 54 kDa fragment (4 h of incubation). Although less perceptible, the same cleavage fragment could be detected in the presence of MntC, used as a positive control, and previously shown to bind human plasminogen (Salazar et al., 2014). It is worth mentioning that the anti-human fibrinogen used in this assay cross-reacted with uPA, which co-migrates with the fibrinogen α-chain. In the absence of uPA (Figure 5, last lane), a faint band corresponding to the fibrinogen α-chain could be observed. Regarding vitronectin, the 75 kDa isoform was almost completely degraded after 1 h of incubation (Figure 6), yielding typical 61–63 kDa fragments reported by Kost et al. (1996). Neither fibrinogen nor vitronectin were cleaved in the absence of uPA or plasminogen (internal control of the reactions). For both fibrinogen and vitronectin, no cleavage fragments were detected in the presence of BSA, used as a negative control protein, since it does not bind plasminogen.

### *aap*-Positive *E. coli* Strains Isolated From Bacteremia Produce Dispersin *in vitro*

The frequency of *E. coli* strains isolated from human bloodstream carrying the dispersin-encoding gene (*aap*) was determined by the amplification of a 232-pb *aap* specific fragment. Among the 278 strains tested, *aap* was found in 8 (2.9%) strains. Sequencing of these amplicons showed that the predict amino acid sequences presented ≥91.1% of identity between them (Supplementary Figure S3). The sequences of these amplicons were deposited in GenBank (accession numbers MT386517, MT386518, MT386519, MT386520, MT386521, MT386522, MT386523, and MT386524). It is worth mentioning that dispersin amino acid sequences of prototype EAEC strains 17-2 and 042 present some changes (L39Q, P51S, K74N, and S109R) although displaying high identity (96.5%). As the dispersin binding motifs were not determined, we cannot exclude that the binding characteristics of dispersin based on the sequence of EAEC 17-2 may differ from other dispersin sequences.

These 8 *aap*-positive strains were further evaluated for the presence of the dispersin secretion system (*aat*), its regulator (*aggR*), the *aai* chromosomal island and the pilin-encoding genes of AAF/I, II, III, IV, and V. Based on that search three of these *aap*-positive strains (EC092, EC194, and EC285) were genetically classified as EAEC, since they concurrently harbored the genes considered as EAEC-defining markers, i.e., *aggR*, *aatA*, and *aaiA*/*aaiG* (Andrade et al., 2014; Robins-Browne et al., 2016). AAF/I (*aggA*) was detected in one of these strains (EC285) and AAF/V (*agg5A*) in two strains (EC194 and EC209), one of them lacking the EAEC defining genes (EC209). We also investigated the adherence pattern on HEp-2 cells displayed by the 8 *aap*-positive and detected the aggregative adherence (Nataro et al., 1987) in 7 of them using the 6-h adherence assay (Table 2 and Supplementary Figure S4). In one strain negative for the presence of EAEC-defining genes (EC206), the adherence pattern was classified as undefined (UND). As these strains were isolated from the bloodstream, the presence of genes encoding ExPEC virulence factors (adhesins, invasins, protectins, iron acquisition systems, and toxins) was assessed (Santos, 2013; Freire et al., 2020). As described in Table 2, they presented distinct and complex ExPEC genes combinations, except for strain EC255 harboring only *iroN* (Table 2).

**TABLE 2:** Phenotypic and genotypic characteristics of *aap*-positive *E. coli* strains isolated from blood.

Strain	Dispersin production^a^	EAEC genes				Adherence pattern^e^	ExPEC genes^f^
		*aat*^b^	*aggR*	*aai*^c^	AAF^d^		
**EC092**	**+**	**+**	**+**	**+**	**−**	AA	*mat*, *iha*, *hra*, *ompA*, *traT*, *irp2*, *iucD*, *sat*
**EC285^g^**	**+**	**+**	**+**	**+**	*aggA* (AAF/I)	AA	*mat*, *iha*, *ompA*, *kpsMT* II, *sitA*, *iucD*, *iroN*, *sat*, *hly*
**EC209^g^**	**+**	**−**	**−**	**−**	*agg5A* (AAF/V)	AA	*mat*, *fimA*, *papA*, *papC*, *iha*, *hra*, *ompA*, *ompT*, *kpsMT* II, *traT*, *sitA*, *irp2*, *chuA*, *iucD*, *ireA*, *hlyA*, *cnf1*
**EC298^g^**	**+**	**−**	**−**	**−**	**−**	AA	*mat*, *fimA*, *afaBCIII*, *ompA*, *kpsMT* II, *chuA*
**EC194^g^**	**−**	**+**	**+**	**+**	*agg5A* (AAF/V)	AA	*mat*, *fimA*, *ompA*, *ompT*, *kpsMT* II, *sitA*, *irp2*, *iucD*
**EC007**	**−**	**−**	**−**	**−**	**−**	AA	*mat*, *fimA*, *ompA*, *kpsMT* II, *traT*, *sitA*, *chuA*
**EC206**	**−**	**−**	**−**	**−**	**−**	UND	*mat*, *fimA*, *ompA*, *ompT*, *kpsMT* II, *traT*, *cvaC*, *sitA*, *irp2*
**EC255**	**−**	**−**	**−**	**−**	**−**	AA	*iroN*

*^a^Dispersin production detected by ELISA. ^b^Results considering the presence of all genes composing the operon: aatA, aatB, aatC, aatD, and aatP. ^c^Results considering the presence of aaiA and aaiG. ^d^AAF, aggregative adherence fimbriae I, II, III, IV, and V. The following pilin-encoding genes were searched: aggA, aafA, agg3A, agg4A, and agg5A. ^e^Adherence pattern observed in HEp-2 cells after 6 h of bacteria-cells incubation: AA, aggregative adherence pattern (Nataro et al., 1987), i.e., bacteria adherent to the HEp-2 cells and coverslip in a stacked-brick configuration; UND, undefined adherence pattern. ^f^Thirty ExPEC virulence factors were searched by DNA-DNA hybridization and PCR: adhesins and/or invasins (mat, fimA, papA, papC, iha, hra, tsh, sfaDE, afaBCIII, bmaE, clpG, ompA, and ibeA), protectins (ompT, kpsMT II, kpsMT III, traT, and cvaC), iron acquisition system (sitA, irp2, chuA, iucD, iroN, and ireA) and toxins (vat, sat, hlyA, hlyF, ehx, and cnf1). The presence or absence of these genes were obtained from Freire et al. (2020) and Santos (2013). ^g^Strains EC194, EC209, EC285 and EC298 were classified as ExPEC by the criteria of Johnson et al. (2003), i.e., the presence of ≥2 of the following genes: papA and/or papC, kpsMT II, afa/dra, sfa/foc, and iucD/iutA.*

**FIGURE 7:**
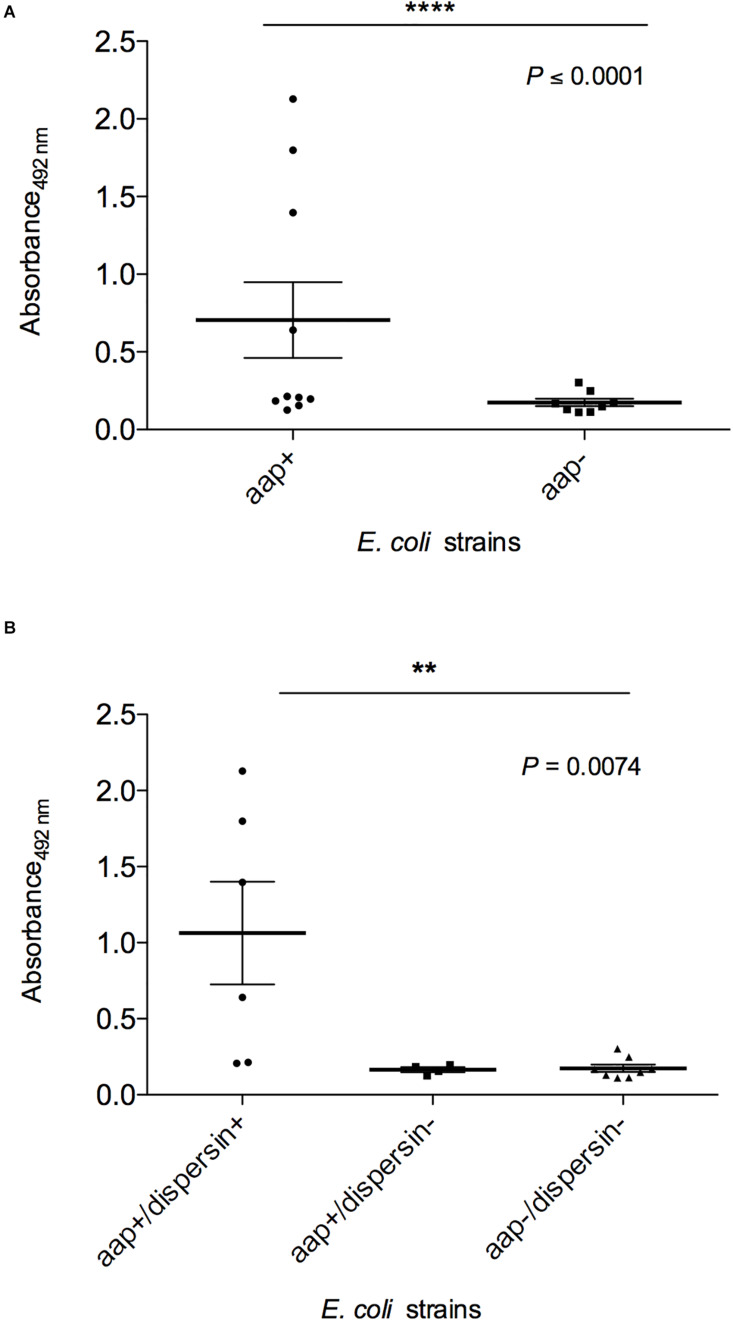
Detection of dispersin by ELISA in *E. coli* strains isolated from blood of human patients. Bacterial pellets of overnight cultures in LB broth were used for coating of ELISA plates, followed by incubation with anti-dispersin polyclonal serum (1:200) produced in rabbit. After washing, horseradish peroxidase-conjugated goat anti-rabbit IgG (Sigma-Aldrich) was added (1:1,000). Blank wells were performed in absence of anti-dispersin sera. EAEC 17-2 and 042 were considered as the positive controls. Data represent the mean absorbance value at 492 nm ± the standard deviation of three independent experiments, performed in triplicates. **(A)** Groups *aap*-positive and *aap*-negative were analyzed by non-parametric Student’s *t*-test (****P ≤ 0.0001) with 95% confidence interval: –0.02416 to 1.086. **(B)** Differences of dispersin production between *aap-*positive strains were analyzed by ANOVA test (**P = 0.0074) with 95% confidence intervals: *aa*p+/dispersin+: 0.1963–1.932; *aap*+/dispersin–: 0.153–0.2157; *aap*–/dispersin–: 0.1171–0.2311.

Finally, dispersin production was evaluated by ELISA in these 8 *aap*-positive strains in comparison to *aap*-negative strains and controls (Figure 7 and Table 2), detecting four significantly (*P* < 0.0001) positive strains: EC092, EC209, EC285, and EC298. These four dispersin-producing strains displayed the AA adherence pattern on HEp-2 cells, but only two of them presented the EAEC-defining genes (EC092 and EC285). It is interesting to note that production of dispersin was detected in two *aap*-positive strains (EC092 and EC285) lacking its regulator and transporter system (*aggR* and *aat*, respectively). On the other hand, dispersin production was not detected in one *aap*-positive strain harboring *aggR* and *aat* (EC194).

## Discussion

In this study, we showed that dispersin can bind to laminin and plasminogen, and dispersin-bound plasminogen is converted into active plasmin in the presence of uPa, which in turn cleaves vitronectin and fibrinogen. The binding of EAEC 042 to laminin has been previously shown, identifying AafA, the major pilin subunit of AAF/II, as a laminin ligand (Farfan et al., 2008; Izquierdo et al., 2014). As AafA and dispersin are exposed on the bacterial surface, our data suggest that laminin binding could be a combined feature of both proteins, at least in strains expressing AAF/II and dispersin. However, AAF/II is found in low frequencies in EAEC collections, while dispersin is highly frequent (Czeczulin et al., 1999; Elias et al., 2002; Jenkins et al., 2007; Boisen et al., 2012; Havt et al., 2017; Hebbelstrup Jensen et al., 2017; Dias et al., 2020). Therefore, our data further demonstrate that dispersin could also contribute to EAEC binding to laminin, which is the major component of the basement membrane (BM) that separates epithelial cells from the underlying stromal cells (Durbeej, 2010). Intestinal pathogens can reach the BM during inflammation or opening of tight junctions, where ECM components are accessible for binding (Kolachala et al., 2007; Strauman et al., 2010; Petrey and de la Motte, 2017). Therefore, we can speculate that the interaction between dispersin and laminin may play a role in bacterial colonization and dissemination, since laminin is present in the intestine and the urinary tract, tissues that can be colonized by EAEC (Nataro et al., 1996; Olesen et al., 2012; Herzog et al., 2014; Nunes et al., 2017; Rajan et al., 2018).

In addition to laminin, dispersin displayed high affinity for plasminogen, a 90-kDa plasma protein produced mainly in the liver, which can be converted into plasmin by tissue and urokinase plasminogen activators (Lähteenmäki et al., 2001). Purified dispersin interacted with plasminogen in a dose-dependent and saturable manner, indicating the specificity of the binding, with an equilibrium dissociation constant (*K*_*D*_) of 0.282 × 10^–6^ M. The assays to characterize the binding nature using the recombinant protein demonstrated that this interaction does not depend on the lysine residues. Although, plasminogen has five Kringle domains (K1–K5) with affinity for C-terminal lysine residues (Lijnen and Collen, 1995; Bhattacharya et al., 2012), which is present in dispersin active form (Velarde et al., 2007). Alternative mechanisms of plasminogen-binding involving arginine and histidine residues have already been described for group A streptococcal M protein (PAM)-related protein – Prp (Sanderson-Smith et al., 2006, 2007). Therefore, we can speculate that dispersin can also bind to plasminogen by different mechanisms, involving other amino acid residues and charge or conformational interactions.

Dispersin-bound plasminogen was converted in active plasmin in the presence of plasminogen activator uPa, as evidenced by degradation of fibrinogen and vitronectin (Figures 5, 6). Due to its proteolytic activities, plasmin plays important roles in pathogenesis when acquired on the bacterial surface, mediating invasion, fibrinolysis, and immune evasion (Lähteenmäki et al., 2005; Peetermans et al., 2016). Gram-negative bacteria including *Haemophilus influenzae*, *Salmonella typhimurium*, *Yersinia pestis*, *Borrelia burgdorferi*, *Leptospira interrogans*, and *Acinetobacter baumannii* use plasminogen as a means to disseminate in host tissues (Lähteenmäki et al., 2001; Sanderson-Smith et al., 2012; Castiblanco-Valencia et al., 2016; Koenigs et al., 2016; Peetermans et al., 2016; Abreu and Barbosa, 2017).

The ability of microorganisms to degrade ECM has a fundamental role on bacterial migration to different sites of infection (Chagnot et al., 2012). Consequently, the multiplicity of proteins on the bacterial surface responsible for ECM degradation is strategic for dissemination. Here, we showed vitronectin cleavage by dispersin-bound plasmin. Vitronectin, a multifunctional protein, circulates in the blood acting as complement regulatory protein, but is also found in the ECM and in a variety of tissues (reviewed in Singh et al., 2010). It harbors multiple domains allowing interaction with several ligands including glycosaminoglycans and collagens (Stockmann et al., 1993; Preissner and Seiffert, 1998). We anticipate that vitronectin degradation by dispersin-bound plasmin may also facilitate *E. coli* dissemination thus contributing to its virulence arsenal.

Data collected from sporadic cases, outbreaks and volunteer studies indicate that bloody diarrhea is a rare symptom associated with EAEC infections (reviewed in Estrada-Garcia et al., 2014 and Hebbelstrup Jensen et al., 2014). However, there are evidences of tissue invasion by EAEC strains detected in cultured epithelial cells (T84, Caco-2 and Int407) and human colonic mucosa (Benjamin et al., 1995; Abe et al., 2001; Pereira et al., 2008; Andrade et al., 2011; Sobieszczańska et al., 2011). Our data do not implicate dispersin as an invasin, promoting entry during the initial stage of infection, but as a protein mediating plasmin presentation on the bacterial surface, which in turn leads to ECM cleavage resulting in tissue spread.

The *aap* gene has already been detected in different *E. coli* pathotypes (Abe et al., 2008; Monteiro et al., 2009; Crossman et al., 2010; Nazemi et al., 2011; Srikhanta et al., 2013; Riveros et al., 2017). In one study the *aap* gene was detected in 46.6% of uropathogenic *E. coli* (UPEC) strains (Nazemi et al., 2011). As bacteremia is frequently a consequence of complicated urinary tract infections, due to bacteria crossing tubular epithelial cell barriers (Flores-Mireles et al., 2015), the presence of dispersin in UPEC may represent an additional virulence factor playing a role in plasmin formation and clot disruption. In fact, we demonstrated that plasmin bound to purified dispersin can cleave fibrinogen (Figure 5). A case of sepsis caused by an *aap*-harboring EAEC strain has been reported, in which the infection evolved from the urinary tract to the bloodstream (Herzog et al., 2014). Considering that dispersin could be involved in the capacity of *E. coli* strains to disseminate in tissues and bloodstream, we searched for the presence of *aap* in a collection of *E. coli* strains isolated from patients with bacteremia, detecting 8 (2.9%) *aap*-positive strains. Although found in low frequency in our strains isolated from the bloodstream, dispersin-positive strains isolated from feces are very frequent (Boisen et al., 2012; Durand et al., 2016; Havt et al., 2017; Hebbelstrup Jensen et al., 2017; Dias et al., 2020). Therefore, the attributes of binding to laminin and degrading ECM may represent a more significant role for dispersin as virulence factor in localized rather than systemic infections.

Four of these *aap*-positive strains produced dispersin *in vitro*, which could be detected even in the absence of the dispersin transporter system (*aatPABCD*) present in EAEC 042, which can suggest that there are alternative mechanisms for dispersin regulation and secretion as suggested by Nishi et al. (2003). Besides AggR, RalR was described as the dispersin regulator in rabbit-specific enteropathogenic *E. coli* (Srikhanta et al., 2013), and CfaD/Rns as the regulator of CexE, a dispersin-like protein of enterotoxigenic *E. coli* (Pilonieta et al., 2007; Crossman et al., 2010). Interestingly, three of the 8 *aap*-positive *E. coli* isolated from bacteremia (EC92, EC194, and EC285) could be categorized as typical EAEC, since they display the AA pattern on HEp-2 cells and harbor *aggR*, *aatPABCD*, and *aaiA*/*aaiG* (Kaper et al., 2004; Robins-Browne et al., 2016). Some studies have reported EAEC as the agent of bacteremia (Telli et al., 2010; Herzog et al., 2014; Lara et al., 2017; Riveros et al., 2017), and the dispersin-encoding gene was present in some of these strains. The presence of at least two genes among *papA* and/or *papC*, *kpsMT* II, *afa*/*dra*, *sfa*/*foc*, and *iucD*/*iutA* indicates that an *E. coli* strain can be classified as ExPEC and has the potential to cause disease in a healthy individual (Johnson et al., 2003). Using this criterion, four strains were classified as ExPEC (EC194, EC209, EC285, and EC298), although EC194 and EC285 were categorized as typical EAEC, suggesting their classification as heteropathogens (Abe et al., 2008; Scheutz et al., 2011; Lara et al., 2017; Gati et al., 2019).

**FIGURE 8:**
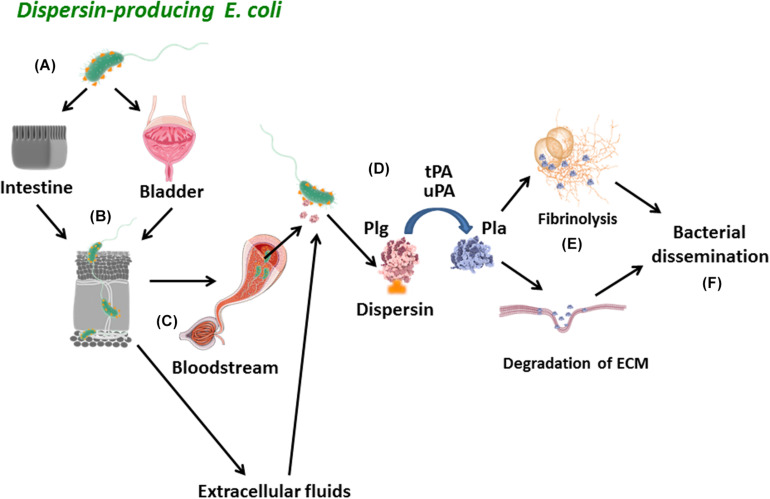
Schematic model of the proposed role for dispersin in bacterial dissemination. **(A)** Dispersin-producing *E. coli* reaches the intestinal or urinary tracts adhering to those epithelia using diverse mechanisms, including binding to laminin; **(B)** Those bacteria presenting invasiveness capacity may penetrate the epithelia getting in contact with connecting tissues, microvessels and the bloodstream; **(C)** In these sites, dispersin exposed on the bacterial surface can bind to Plasminogen; **(D)** Plasminogen bound to Dispersin can be activated into Plasmin in the presence of plasminogen activators (uPa and tPa); **(E)** Active Plasmin can act as a potent protease promoting degradation of physiologic subtracts such as fibrin clots and/or extracellular matrix proteins; **(F)** These two effects lead to bacterial dissemination. Plg, plasminogen; Pla, plasmin; uPa, urokinase plasminogen activator; tPa, tissue plasminogen activator; ECM, extracellular matrix proteins.

In conclusion, we described here new attributes of dispersin by its interaction with laminin and plasmin(ogen). A proposed schematic model for the role of dispersin in the pathogenesis of *E. coli* is depicted in Figure 8. Dispersin-producing *E. coli* reaches the intestinal or urinary tracts adhering to those epithelia using diverse mechanisms, including the binding to laminin. Those bacteria presenting invasiveness capacity may penetrate the epithelia getting in contact with connecting tissues, microvessels and the bloodstream, where dispersin exposed on the bacterial surface can bind to plasminogen. Then, dispersin-bound plasminogen can be activated into plasmin in the presence of plasminogen activators, acting as a protease promoting degradation of ECM components or fibrin clots, leading to bacterial dissemination. Nevertheless, our data were generated using purified dispersin and further studies are necessary to address the contribution of dispersin in tissue adhesion/dissemination using dispersin-producing *E. coli*.

## Data Availability Statement

The raw data supporting the conclusions of this article will be made available by the authors, without undue reservation, to any qualified researcher.

## Ethics Statement

The animal study was reviewed and approved by the Ethics Committee on Animal Use of the Butantan Institute (CEUAIB Protocol #731408317).

## Author Contributions

CM, AB, and WE contributed to conception and design. CM, JL, LS, DP, EC, MM, NR, SS, RP, AB, and WE were responsible for the acquisition and analysis. CM, JL, LS, DP, EC, MM, NR, SS, AS, RP, AB, and WE worked on the interpretation and had substantial contributions. CM, JL, DP, EC, AS, RP, AB, and WE drafted the work or revised it critically. CM, AB, and WE were responsible for the final approval of the version to be published.

## Conflict of Interest

The authors declare that the research was conducted in the absence of any commercial or financial relationships that could be construed as a potential conflict of interest.

## Supplementary Material

FIGURE S1 Biochemical characterization of recombinant dispersin. **(A)** C18-RP-HPLC separation of the filtered culture medium. The arrow indicates the recombinant dispersin. Insert: MALDI-TOF/MS profile of the manually collected peak, indicating homogeneity in the 2000–18000 m/z range. **(B)** Direct infusion ESI-IT-TOF/MS profile of the purified recombinant dispersin. The multiply charges ions are indicated and the calculated molecular mass is printed.

FIGURE S2 Circular dichroism spectrum and resulting deconvolution analysis. Dispersin was dialyzed on 10 mM phosphate buffer and, then, circular dichroism spectra were collected at 25°C. The spectrum shown is an average of 10 scans. The deconvolution analysis indicates that the main secondary structure of the recombinant dispersin is beta-sheet.

FIGURE S3 Alignment of amino acid sequences of dispersin of *aap*-positive *E. coli* strains. Amino acid sequences were aligned using BioEdit vs. 7.0.5 to compare the prototype EAEC strains (17-2 and 042) and *aap*-positive *E. coli* strains isolated from bacteremia. The identity between the samples was ≥91.1%. Note the amino acid changes between EAEC 17-2 and 042 sequences: L39Q, P51S, K74N and S109R.

FIGURE S4 Adherence pattern on HEp-2 cells displayed by the *aap*-positive *E. coli* strains. The aggregative adherence (AA) pattern was observed on HEp-2 cells after 6 h of incubation with strains EC007, EC092, EC194, EC209, EC255, EC285, and EC298, while strain EC206 presented an undefined (UND) adherence pattern. The coverslips were observed by light microscopy (1,000 X). Strains EAEC 042, EAEC 17-2 and *E. coli* DH5α were included as controls for AA (042 and 17-2) and non-adherence (DH5α), using the 3-h incubation assay.
