# Supplementary figures and images for: Surface Protein Dispersin of Enteroaggregative Escherichia coli Binds Plasminogen That Is Converted Into Active Plasmin
Source: Front Microbiol. 2020 Jun 18;11:1222. doi: 10.3389/fmicb.2020.01222 (PMC7315649; doi:10.3389/fmicb.2020.01222)

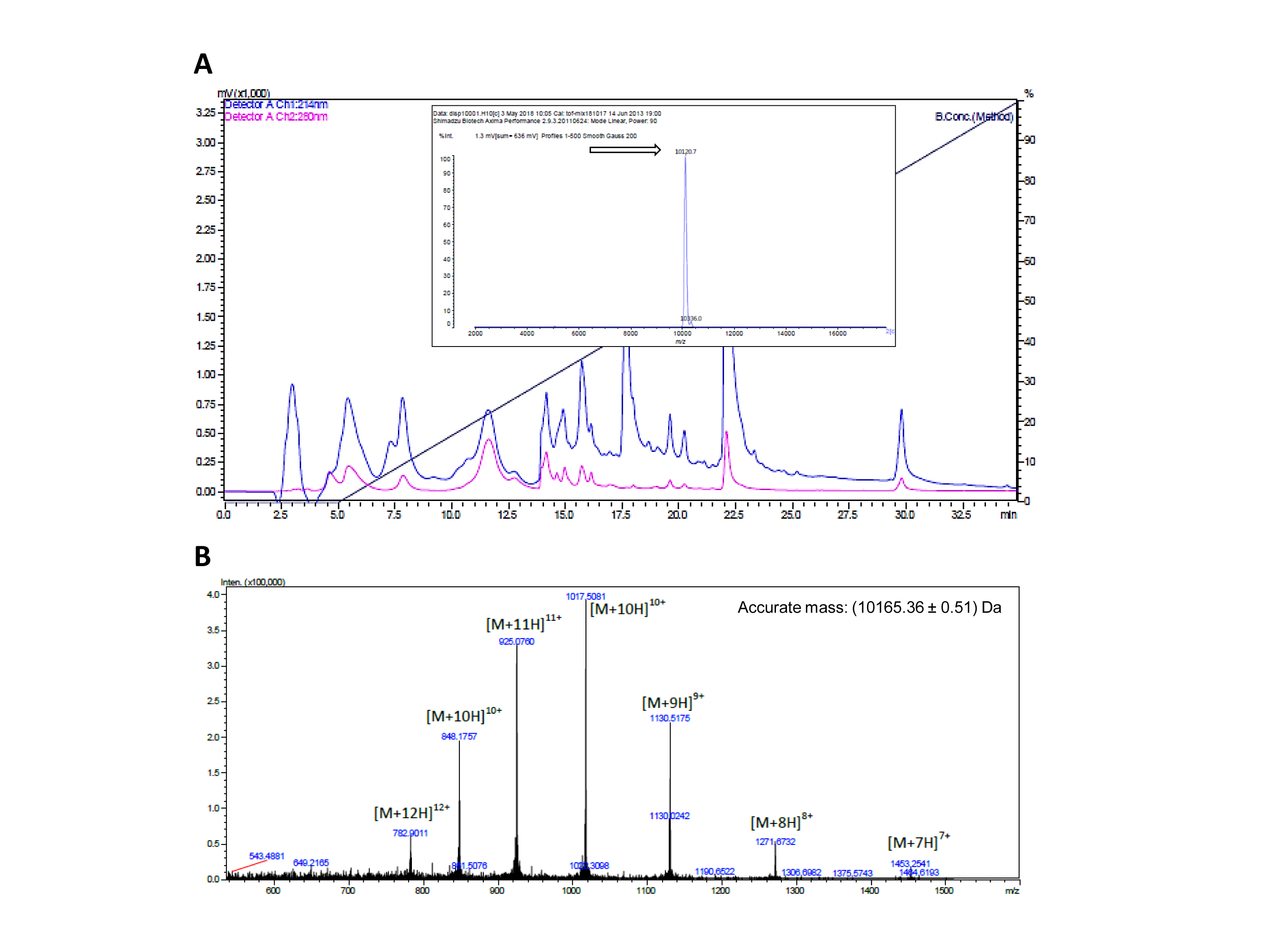

Supplement: FIGURE S1 — Biochemical characterization of recombinant dispersin. (A) C18-RP-HPLC separation of the filtered culture medium. The arrow indicates the recombinant dispersin. Insert: MALDI-TOF/MS profile of the manually collected peak, indicating homogeneity in the 2000–18000 m/z range. (B) Direct infusion ESI-IT-TOF/MS profile of the purified recombinant dispersin. The multiply charges ions are indicated and the calculated molecular mass is printed. [file Image_1.TIF]

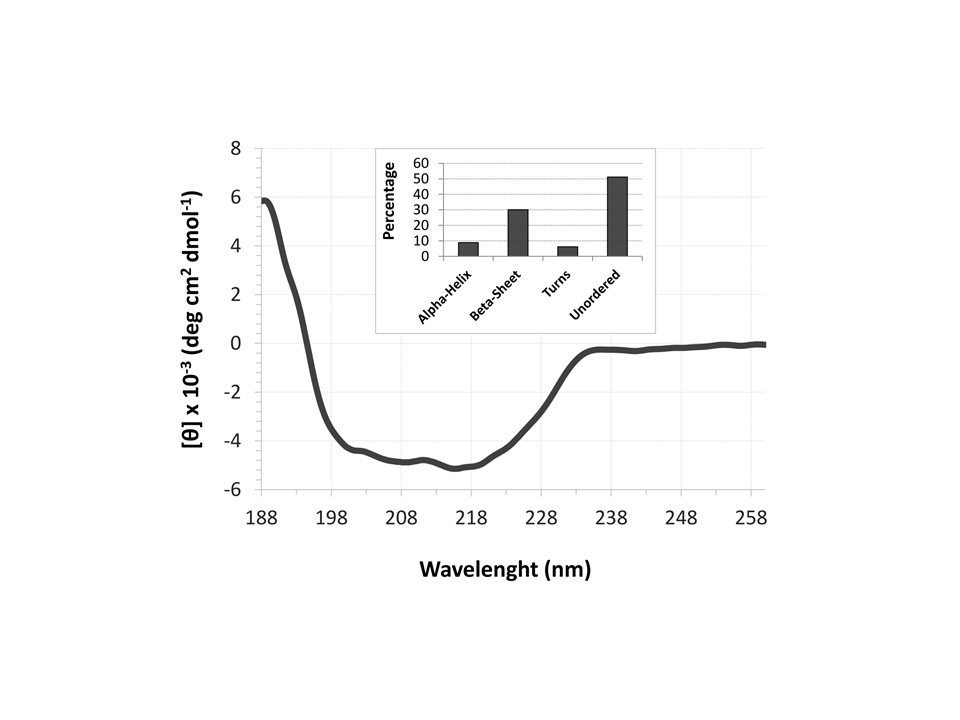

Supplement: FIGURE S2 — Circular dichroism spectrum and resulting deconvolution analysis. Dispersin was dialyzed on 10 mM phosphate buffer and, then, circular dichroism spectra were collected at 25°C. The spectrum shown is an average of 10 scans. The deconvolution analysis indicates that the main secondary structure of the recombinant dispersin is beta-sheet. [file Image_2.TIF]

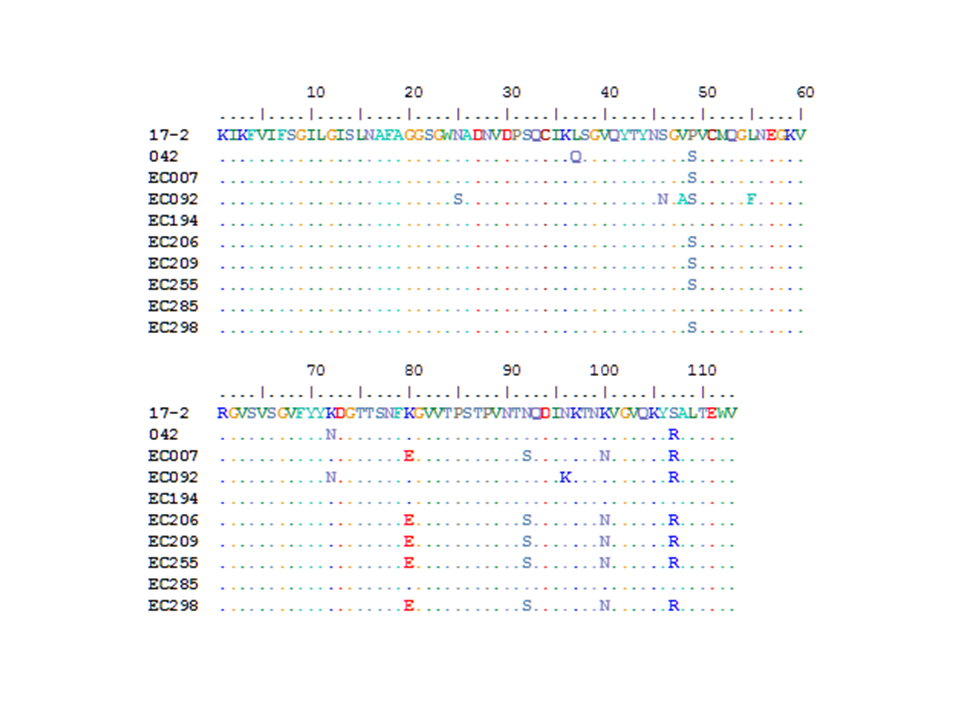

Supplement: FIGURE S3 — Alignment of amino acid sequences of dispersin of aap-positive E. coli strains. Amino acid sequences were aligned using BioEdit vs. 7.0.5 to compare the prototype EAEC strains (17-2 and 042) and aap-positive E. coli strains isolated from bacteremia. The identity between the samples was ≥91.1%. Note the amino acid changes between EAEC 17-2 and 042 sequences: L39Q, P51S, K74N and S109R. [file Image_3.TIF]

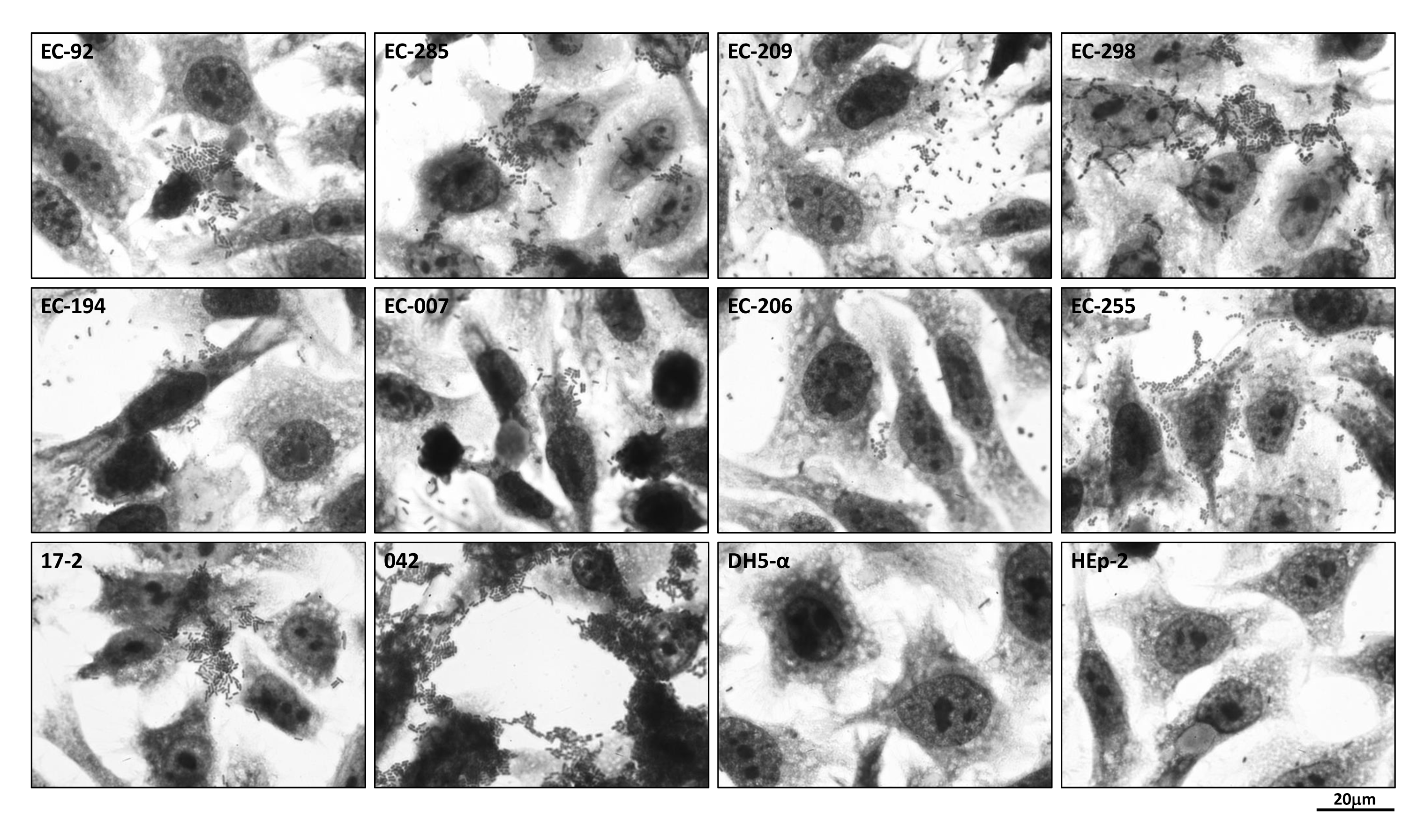

Supplement: FIGURE S4 — Adherence pattern on HEp-2 cells displayed by the aap-positive E. coli strains. The aggregative adherence (AA) pattern was observed on HEp-2 cells after 6 h of incubation with strains EC007, EC092, EC194, EC209, EC255, EC285, and EC298, while strain EC206 presented an undefined (UND) adherence pattern. The coverslips were observed by light microscopy (1,000 X). Strains EAEC 042, EAEC 17-2 and E. coli DH5α were included as controls for AA (042 and 17-2) and non-adherence (DH5α), using the 3-h incubation assay. [file Image_4.TIF]
